# A model to simulate human cardio-respiratory responses to ketamine analgesia

**DOI:** 10.3389/fphys.2025.1654768

**Published:** 2025-11-13

**Authors:** Varghese Kurian, Xin Jin, Anders Wallqvist, Jaques Reifman, Sridevi Nagaraja

**Affiliations:** 1 Department of Defense Biotechnology High Performance Computing Software Applications Institute, Defense Health Agency Research & Development, Medical Research and Development Command, Fort Detrick, MD, United States; 2 The Henry M. Jackson Foundation for the Advancement of Military Medicine, Inc., Bethesda, MD, United States

**Keywords:** cardiovascular system, combat casualty, mathematical model, hemorrhage, pharmacokinetic-pharmacodynamic, pain management

## Abstract

In large-scale combat operations, decision-support systems based on artificial intelligence technology can augment the capability and capacity of medics to care for combat casualties. The development and assessment of such systems require large amounts of vital-sign data, which are impractical to obtain from clinical or experimental studies but can be generated using high-fidelity computational models. We previously developed and validated a human cardio-respiratory model (the CR model) that accounts for both cardiovascular and respiratory responses to hemorrhagic injuries and resuscitation with six commonly used fluid types. Here, we extended the CR model to represent the effect of ketamine analgesia on the vital signs by integrating two existing pharmacokinetic-pharmacodynamic models of ketamine [one representing the effect of ketamine on mean arterial pressure (MAP) and another on cardiac output], with two feedback controllers described within the CR model. We validated the extended model using experimental data of ketamine administration in three human studies for healthy individuals. Simulation results showed that the model captured the general trend of the experimental data for MAP and heart rate (HR) with root mean square errors of 6.17 mmHg and 7.51 beats/min, respectively. In addition, in simulations of hemorrhagic injury, ketamine administration rapidly increased MAP and cardiac output, but the magnitude of the increase decreased with hemorrhage severity. MAP increased by 40%, 30%, and 15% of its baseline value in the presence of no, moderate, and severe hemorrhage, respectively. Given that most wounded Service members receive pain medication in the form of ketamine, the ability to account for its effects on vital signs will allow us to generate more comprehensive synthetic data of combat casualties.

## 1 Introduction

Pain management is a central aspect of battlefield trauma care (Buckenmaier and Griffith, 2010). Recent analyses of U.S. combat casualties have reported that over 70% of injured Service members received some form of pain medication near the point of injury (POI) or during tactical evacuation (Shackelford et al., 2015; Schauer et al., 2017). In future large-scale combat operations with a near-peer adversary, we anticipate high casualty rates and significant delays in casualty evacuation in a contested airspace (Dolan et al., 2021). In this scenario, where trauma casualties are expected to be treated in the field for prolonged periods of time, the use of analgesics will be a crucial component in pain management. By alleviating pain, analgesics curb a plethora of other pain-induced responses, such as nausea, vomiting, urinary retention, and impaired immune function, while increasing casualty cooperation, which in turn improves treatment outcomes and can potentially reduce the risk of post-traumatic stress disorder (Thomas and Shewakramani, 2008; Buckenmaier and Griffith, 2010; Shackelford et al., 2015). However, analgesics are also known to affect cardiovascular responses, such as blood pressure and heart rate (HR) (Miller et al., 2016), which can be substantially affected in hemorrhagic injuries, the primary cause of death on the battlefield (Katzenell et al., 2012). As such, combat medics must consider these effects when administering analgesics to hemorrhagic casualties during prolonged care.

Clinical decision-support systems that leverage artificial intelligence (AI) and machine-learning (ML) algorithms can augment the capability and capacity of combat medics to monitor, triage, and treat combat casualties near the POI (Jin et al., 2018; Craca et al., 2023; Peng et al., 2023; Stallings et al., 2023; Jin et al., 2024). However, to develop such AI and ML algorithms, we require massive amounts of curated data from a wide range of injury and treatment scenarios, which are challenging to obtain from existing animal studies due to their small sample sizes (<50 subjects) or from clinical studies, which typically record only a few variables (Shaked et al., 2009; Cosgriff et al., 2019; de Rocquigny et al., 2020). A viable alternative is to use validated physiological models to generate synthetic data of battlefield injury and treatment scenarios and then use the data to train and assess the performance of these algorithms.

Over the last 30 years, a number of mechanistic models of the cardio-respiratory system have been developed to predict the effect of hemorrhagic injuries and fluid resuscitation on vital signs (Mardel et al., 1995; Drobin and Hahn, 1999; Tatara et al., 2007; Curcio et al., 2021; Jin et al., 2023; Kurian et al., 2025). However, none of these models accounts for the effect of analgesics on the dynamics of the cardio-respiratory system because their mechanisms are not well understood. An alternative approach is to develop data-driven, pharmacokinetic-pharmacodynamic (PK-PD) models trained on large amounts of clinical and experimental data and fitting them using empirical relationships. These models have been shown to adequately predict the effects of different analgesics, including ketamine and fentanyl, on the physiological responses of vital signs (Yassen et al., 2007; Dahan et al., 2011; Kamp et al., 2021; Abuhelwa et al., 2022). However, due to their dependence on data, PK-PD models are often limited to characterize physiological responses for a specific population or disease condition. Clipp et al. (2016) overcame this limitation by using PK-PD models to predict the effects of different drugs, including ketamine, on vital signs and then incorporating them into a mechanistic cardiovascular model of hemorrhagic injury as fractional increases or decreases relative to their baseline values without the drug. However, their model [the Pulse Physiology Engine (Bray et al., 2019)] is complex, involving thousands of parameters and variables, and has largely only been validated by qualitative comparisons with experimental data. To date, quantitatively validated physiological models that consider both the dynamics of the human cardio-respiratory system and the effect of ketamine on vital signs are not available.

Recently, we developed the cardio-respiratory (CR) model to capture human physiological responses to hemorrhage, respiratory perturbations, and associated fluid treatments with six different fluid types (Jin et al., 2023; Kurian et al., 2025). We validated the model’s predictions of vital signs and blood variables, including mean arterial pressure (MAP), HR, cardiac output (CO), end-tidal carbon dioxide concentration, hemoglobin concentration, and delivered oxygen, using both human and animal data involving a wide range of hemorrhagic injuries and respiratory perturbations. We later used this model to generate synthetic data for the training and validation of an AI algorithm that can assist combat medics in allocating fluids to casualties in a resource-constrained environment (Jin et al., 2024). Here, we extended the CR model to incorporate the effects of ketamine, the recommended analgesic for combat casualties with hemorrhagic injury (Committee on Tactical Combat Casualty Care, 2024), on the vital signs, including MAP, CO, and HR. Specifically, we used the outputs from two existing PK-PD models of ketamine (one PD model predicted the effect of ketamine on MAP and the other on CO) and integrated them into the CR model by modifying the implementation of two feedback controllers. Based on the MAP and CO changes, the CR model also predicted the corresponding changes in HR. We validated the extended model by comparing its predictions with data from three human studies not used for model calibration, involving bolus ketamine administration in healthy individuals. Finally, we used the model to simulate the effects of ketamine on the vital signs of individuals suffering from a hemorrhagic injury, a scenario that has rarely been addressed in prior computational or experimental studies (Xiang et al., 2020; Klemcke et al., 2021).

## 2 Methods

### 2.1 Computational model

**FIGURE 1:**
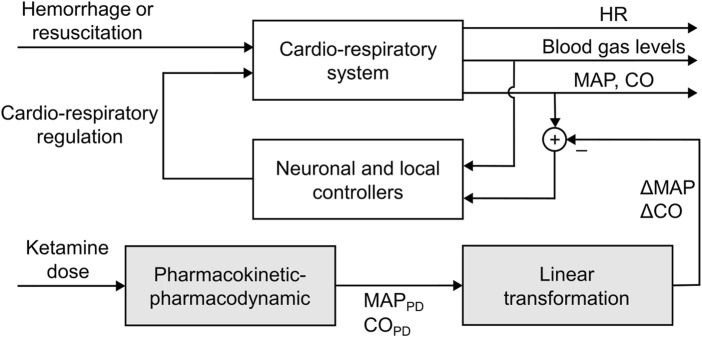
Schematic showing the integration of the pharmacokinetic-pharmacodynamic model into the cardio-respiratory model, with the shaded boxes indicating the newly added extensions. CO, cardiac output; HR, heart rate; MAP, mean arterial pressure.

We previously developed the CR model to predict the temporal changes in vital signs following mild to severe hemorrhagic injury and subsequent fluid treatment (Jin et al., 2023; Kurian et al., 2025). The CR model represents the cardiovascular and respiratory systems and their regulatory mechanisms using a set of 104 ordinary differential and algebraic equations and 118 parameters. Here, we extended this model to incorporate the effects of analgesic doses of racemic ketamine on vital signs, including MAP, HR, and CO. To this end, we added two new components to the CR model: a PK-PD component and a linear transformation component (Figure 1, shaded boxes). Given a dose of ketamine, the PK-PD component determines the resulting values $\text{MA}P_{\text{PD}}$ and $CO_{\text{PD}}$ to be used as inputs to the linear transformation component. This component then calculates the modifications ( ΔMAP and ΔCO ) needed by the neuronal and local controllers as a feedback to drive the CR model predictions of MAP and CO to values that match those determined by the PK-PD component (i.e., so that MAP = $\text{MA}P_{\text{PD}}$ and CO = $CO_{\text{PD}}$ ). For a comprehensive overview of the original CR model formulation and implementation, we direct the reader to Jin et al. (2023) and Kurian et al. (2025).

#### 2.1.1 Ketamine pharmacokinetic-pharmacodynamic model

Racemic ketamine, a mixture of equal amounts of *S*- and *R*-ketamine, is the preferred analgesic for hemorrhagic trauma patients (Committee on Tactical Combat Casualty Care, 2024). Ketamine is known to drive multiple cardiovascular responses in the body, including increases in MAP, HR, and CO (DeLorenzo et al., 2015; Peltoniemi et al., 2016; Vankawala et al., 2021; Mostafa et al., 2024). In the CR model, we incorporated the effect of ketamine on these vital signs through a two-step process. First, we used the PK model developed by Kamp et al. (2020) (comprising of 12 equations and 11 parameters) to calculate the changes in plasma concentrations of ketamine and its metabolite (norketamine) over time when it was administered via intravenous infusion at different doses and rates (Kamp et al., 2020). Second, we used the PD model developed by Abuhelwa et al. (2022) (comprised of 1 equation and 3 parameters) to calculate the ketamine-induced increase in MAP and the PD model developed by Kamp et al. (2021) (comprised of 3 equations and 4 parameters) to calculate the corresponding increase in CO. Using the plasma concentrations of ketamine and norketamine determined by the PK model as inputs to both PD models, they determined the resulting increases in MAP and CO (i.e., $\text{MA}P_{\text{PD}}$ and $CO_{\text{PD}}$ , respectively) as the outputs. Here, we used the same equations and parameter values provided in the original PK-PD models, which were developed using data from healthy individuals absent of pain.

#### 2.1.2 Incorporation of the PK-PD model outputs into the CR model via linear transformation

We used the approach proposed by Magosso and Ursino to incorporate the ketamine PK-PD model-predicted increases in MAP and CO into the CR model (Magosso et al., 2004). Briefly, the existing CR model includes descriptions for neuronal and local feedback controllers that represent the effect of sympathetic stimulation on cardiovascular resistance and the autoregulation of blood flow in the body. The objective of these controllers is to preserve homeostasis, which in the model is defined as maintaining MAP at 100 mmHg and CO at 5 L/min. At each simulation time step, these controllers receive the current values of MAP and CO as inputs, and when they differ from their target homeostasis values, the controllers take specific actions, such as decreasing the arterial resistance or increasing the heart strength, or both, to restore MAP and CO to their respective target values.

To implement the ketamine-induced increases in vital signs, we modified the MAP and CO input values to these controllers such that their subsequent feedback would drive the CR model-predicted MAP and CO to match the corresponding values predicted by the PK-PD models ( $\text{MA}P_{\text{PD}}$ and $CO_{\text{PD}}$ ). Specifically, at each simulation time step, based on the $\text{MA}P_{\text{PD}}$ and $CO_{\text{PD}}$ values provided by the PK-PD models for a given ketamine dose, the linear transformation computed ΔMAP and ΔCO and the model subtracted them from the current CR-model predicted MAP and CO values before providing them as inputs to the controllers (Figure 1, summing point). To determine the time-dependent values of ΔMAP and ΔCO that would induce the appropriate feedback within the CR model’s neuronal and local controllers, we performed 10,000 simulations using the original CR model. In these simulations, we introduced 10,000 unique combinations of ΔMAP (between −20 and 80 at 1-mmHg increments) and ΔCO (between −2 and 4 at 0.17-L/min increments) to modify the input to the controllers in the model and recorded the resultant MAP and CO values at steady state. Using these simulated data, we separately fitted two linear models between ΔMAP and ΔCO and the updated vital signs ( $\text{MA}P_{\text{PD}}$ and $CO_{\text{PD}}$ ) induced by ketamine determined by the PK-PD models, as follows:

$$\Delta \text{MAP}=-217.90\;‐\;1.47\text{ MA}P_{\text{PD}}+14.01\;CO_{\text{PD}}$$ (1)

$$\Delta \text{CO}=-14.68\;‐\;5.28\times 10^{‐3}\text{ MA}P_{\text{PD}}+3.02\;CO_{\text{PD}}$$ (2)

We computed the *R* ^2^ values to assess the goodness-of-fit of the linear models described by these two equations, obtaining an *R* ^2^ of 0.99 for Equation 1 and 0.98 for Equation 2. Because the linear models yielded a good fit, we did not test alternative, nonlinear models. In this procedure, we assumed that the ketamine-driven changes in MAP and CO (in the PK-PD models and Equations 1 and 2) derived using data from healthy individuals hold for individuals who suffered hemorrhagic trauma and received fluid treatment.

Overall, we added 18 new equations and 24 new parameters to the CR model, including the equations and parameters of the PK-PD models and linear transformation. The extended model consisted of 122 ordinary differential and algebraic equations and 142 associated parameters. The model inputs included the rate of hemorrhage, type of resuscitation fluid and its infusion rate, ketamine dose and infusion rate, minute ventilation, and fraction of inspired oxygen. The model outputs included the mean, systolic, and diastolic arterial blood pressures, HR, CO, hemoglobin concentration, oxygen saturation, and partial pressure of end-tidal carbon dioxide. We performed all simulations using MATLAB 24.2 (MathWorks, Natick, MA) and solved the model equations using Euler’s method (Griffiths and Higham, 2010) with a time step of 4.17 × 10^−4^ min.

#### 2.1.3 Sensitivity analysis

We performed a comprehensive extended Fourier amplitude sensitivity testing (eFAST) analysis to identify the contribution of each of the 24 newly added parameters (shown in Table 1) to the variance of the model outputs and to evaluate the model’s robustness to variations in these parameter values, especially those directly adopted from previous models (11 from the PK model and 7 from the PD models). In total, we analyzed 8,760 simulated ketamine administration scenarios. In all scenarios, we performed a 60-min simulation, where at time 0 min we administered 0.25 mg/kg ketamine over 1 min. To choose the parameter values for each simulation, we first defined their feasible ranges as ±20% of their nominal values. We then defined a sinusoidal function of a particular frequency for each of the 24 parameters, sampled each parameter 73 times, and repeated the sampling procedure five times to generate 8,760 parameter sets (i.e., 24 × 73 × 5) for the simulations. At the end of each simulation, we analyzed changes in four model outputs, *S*-ketamine, MAP, HR, and CO, after ketamine administration. Next, using Fourier analysis, for each model output, we estimated 24 sensitivity indices by calculating the variance in the output at a particular parameter’s unique frequency divided by the total variance. The values of these indices fell between 0 and 1, with higher values indicating a stronger contribution of a given parameter to the variance of the output. We performed all simulations and sensitivity calculations using a slightly modified version of a MATLAB code (Nagaraja et al., 2017) previously proposed by Marino et al. (2008).

**TABLE 1:** List of newly added parameters in the extended CR model with their assigned numbers (P#), descriptions, values, and units.

P#	Parameter description	Value^a^	Unit
1	*S*-ketamine elimination clearance	1.78	L/min
2	*R*-ketamine elimination clearance	1.58	L/min
3	Central compartment volume for ketamine	25.80	L
4	Intercompartmental clearance for ketamine	2.10	L/min
5	Peripheral compartment volume for ketamine	115.00	L
6	Peripheral compartment volume for norketamine	240.00	L
7	*S*-norketamine elimination clearance	1.00	L/min
8	*S*-norketamine intercompartmental clearance	3.27	L/min
9	Norketamine mean transit time	26.60	min
10	*R*-norketamine central clearance	0.73	L/min
11	*R*-norketamine intercompartmental clearance	2.55	L/min
12	*S*-ketamine concentration for 25% change in CO	4.00 × 10^−4^	g/L
13	*S*-norketamine concentration for 25% change in CO	1.60 × 10^−4^	g/L
14	*S*-ketamine effect compartment half-life for CO	2.28	min
15	*S*-norketamine effect compartment half-life for CO	29.30	min
16	Maximum effect of ketamine on MAP	51.60	mmHg
17	Ketamine concentration with half-maximal effect on MAP	4.68 × 10^−3^	g/L
18	Exponent in the pharmacodynamic model of MAP	2.04	-
19	Intercept term in linear model of ΔMAP	−217.90	mmHg
20	Coefficient of $\text{MA}P_{\text{PD}}$ in linear model of ΔMAP	1.47	-
21	Coefficient of $CO_{\text{PD}}$ in linear model of ΔMAP	14.01	mmHg⋅min/L
22	Intercept term in linear model of ΔCO	−14.68	L/min
23	Coefficient of $\text{MA}P_{\text{PD}}$ in linear model of ΔCO	−5.28 × 10^−3^	L/(min⋅mmHg)
24	Coefficient of $CO_{\text{PD}}$ in linear model of ΔCO	3.02	-

CO, cardiac output; MAP, mean arterial pressure.

^a^ Parameters 1–11 were obtained from Kamp et al. (2020), Parameters 12–15 from Kamp et al. (2021), and Parameters 16–18 from Abuhelwa et al. (2022). Parameters 19–24 were estimated using linear models.

### 2.2 Model validation

To validate the extended model, we compared its predictions with previously reported experimental data from the three studies shown in Table 2 (Kochs et al., 1991; Krystal et al., 1998; DeLorenzo et al., 2015). Briefly, all three studies (*Studies 1–3*) reported the temporal changes in MAP and HR after an intravenous bolus dose of racemic ketamine ranging from 0.23 to 0.26 mg/kg over 1 min in healthy human participants. In *Studies 1* and *3*, immediately after the bolus, participants also received a ketamine infusion ranging from 0.58 to 0.65 mg/kg at a constant rate over 60 min (Krystal et al., 1998; DeLorenzo et al., 2015). For additional information on the experimental protocols of these studies, we refer the reader to the original articles.

**TABLE 2:** Studies used for validation of the extended cardio-respiratory (CR) model.

Study	No. of subjects	Ketamine dose (mg/kg)		MAP			HR			References
		Bolus (1 min)	Slow (60 min)	RMSE (mmHg)	MBE (mmHg)	%	RMSE (beats/min)	MBE (beats/min)	%	
1	10	0.23	0.58	5.64	2.68	73	3.78	−0.47	100	DeLorenzo et al. (2015)
2	10	0.25	-	6.43	2.05	63	10.05	−6.94	38	Kochs et al. (1991)
3	8	0.26	0.65	6.45	−5.58	100	8.70	−7.24	40	Krystal et al. (1998)

HR, heart rate; MAP, mean arterial pressure; MBE, mean bias error; RMSE, root mean square error.

To simulate the scenarios in *Studies 1–3*, we provided the corresponding ketamine administration dose and rate as an input to the CR model, and the model predicted the time courses of MAP and HR. Then, we compared the simulated time courses of these variables with the corresponding experimental data. Because at baseline the values of the vital signs varied between studies, we normalized the model-predicted outputs before comparing them with the experimental data. To this end, we multiplied each predicted output by the ratio of their experimental to predicted values at baseline (Jin et al., 2023). Finally, we computed the root mean square error (RMSE) and the mean bias error (MBE) between the normalized model predictions and the corresponding experimental data as well as the percentage of the normalized model-predicted values that fell within ±2 standard errors of the mean (SEM) of the experimental data (Table 2).

To assess the uncertainty in the predictions of cardiovascular responses to ketamine administration, in addition to simulating the experimental scenarios in *Studies 1–3* with the nominal parameter set, we also simulated each of the three scenarios 1,000 times using 1,000 unique parameter sets of the PD models (Kamp et al., 2021; Abuhelwa et al., 2022). To generate the parameter sets, we randomly selected the parameter values from within the 95% confidence intervals of their respective means using a Latin hypercube sampling method (Cook et al., 2015). These simulations allowed us to establish the range of MAP and HR responses induced by the same dose of ketamine resulting from uncertainties in the estimation of the PD model parameters.

### 2.3 *In silico* analysis of the effect of hypovolemia

We also used the extended CR model to investigate the cardiovascular responses to ketamine administration during hemorrhagic injury. Because we could not identify previous large-animal studies or human studies involving both hemorrhagic injury and ketamine administration, we simulated two arbitrary scenarios. In the first scenario, we simulated the experimental protocol described in Urbano et al. (2012) after adding ketamine administration following a hemorrhagic injury. This protocol involved swine models challenged with a hemorrhage of ∼30% of their total blood volume and subsequent resuscitation to ∼15% of the total blood volume with a mixture of 5% albumin and 3% hypertonic saline. We compared the model-predicted temporal changes in MAP and cardiac index (CI; CO divided by body surface area) with the experimental data after normalizing them using the procedure described in Section 2.2 “Model validation.” We repeated this simulation with intravenous ketamine administration five times. Starting at 2 min after the end of hemorrhage, we provided the first ketamine infusion (0.25 mg/kg for a 70-kg adult human) and repeated this infusion four more times 25 min apart. Then, we repeated the entire simulation procedure with two other ketamine doses of 0.20 and 0.30 mg/kg. We selected the three ketamine doses (0.20, 0.25, and 0.30 mg/kg), infusion time (1 min), and time interval between two consecutive doses (i.e., 25 min) based on the Tactical Combat Casualty Care guidelines (Committee on Tactical Combat Casualty Care, 2024). In the second scenario, we simulated a hemorrhage of 45% of total blood volume followed by a fluid resuscitation to 23% of the total blood volume with a mixture of 5% albumin and 3% hypertonic saline. In these simulations, we used the same ketamine administration protocol and performed the same normalization procedures and comparisons as in the first scenario.

## 3 Results

### 3.1 Contribution of the model parameters to the variance of the model outputs

**FIGURE 2:**
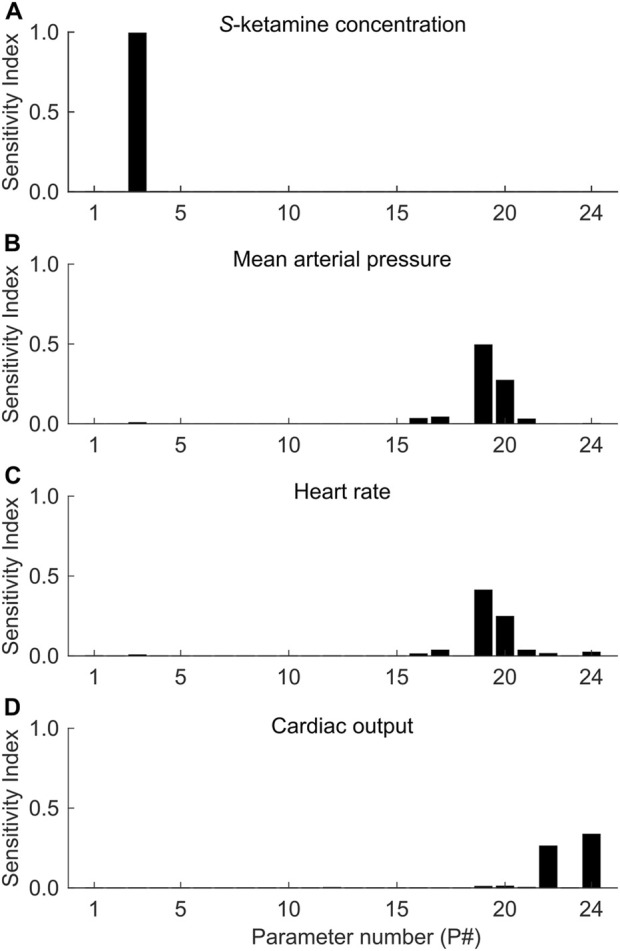
Extended fourier amplitude sensitivity testing (eFAST) sensitivities of the extended cardio-respiratory (CR) model’s outputs **(A)** *S*-ketamine concentration, **(B)** mean arterial pressure, **(C)** heart rate, and **(D)** cardiac output to the 24 newly added model parameters. We show the names and nominal values of the 24 parameters in Table 1.

Figure 2 shows the sensitivity of the four model outputs analyzed (*S*-ketamine, MAP, HR, and CO) after ketamine administration, for each of the 24 newly added model parameters. We found that the parameter representing the central compartment volume (P#3 in Figure 2A) used in the PK model (Kamp et al., 2020) had the highest contribution to the variance of the peak *S*-ketamine concentration with a sensitivity of 0.99. The sensitivities of all the other 23 parameters were <0.01. For both MAP and HR, the parameters of the linear model in Equation 1 representing the intercept term and the $\text{MA}P_{\text{PD}\;}$ coefficient (P#19 and P#20 in Figures 2B,C) considerably contributed to their variance, with sensitivities of 0.49 and 0.27, respectively, for MAP and 0.41 and 0.25, respectively, for HR. Finally, for CO, the parameters of the linear model in Equation 2 representing the intercept term and the $CO_{\text{PD}}$ coefficient (P#22 and P#24 in Figure 2D) largely contributed to its variance with sensitivities of 0.26 and 0.34, respectively. Thus, out of the 24 parameters of the ketamine model, five parameters yielded the largest effects on four model outputs.

### 3.2 Model validation

**FIGURE 3:**
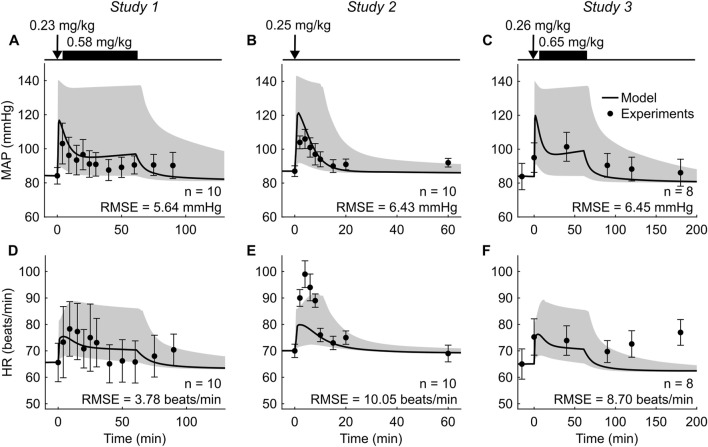
Comparison of the extended cardio-respiratory vital-sign model predictions with the corresponding experimental data from *Studies 1–3*. **(A–C)** Mean arterial pressure (MAP) and **(D–F)** heart rate (HR). The timeline at the top of each panel illustrates the experimental protocol, wherein the arrows represent bolus ketamine and the solid bars denote a slow, 60-min ketamine administration. Shaded areas represent the range of uncertainty in model predictions obtained from 1,000 model simulations, each performed using a different set of pharmacodynamic model parameter values. Error bars denote two standard errors of the mean. RMSE: root mean square error.

To validate the extended CR model, we compared its predictions of MAP and HR (Figure 3, solid lines) with the experimental data in *Studies 1–3* involving healthy individuals (Table 2; Figure 3, solid circles). Overall, the model predictions captured the general trend of the experimental data. For example, within 2 min of ketamine administration, the predicted MAP increased by ∼40% of its baseline values (Figures 3A–C) and HR increased by ∼15% (Figures 3D–F). Both vital signs remained elevated during ketamine administration but returned to their respective baseline values within ∼30 min after ketamine administration ended. On average, across the studies, we obtained a RMSE of 6.17 mmHg for MAP and 7.51 beats/min for HR. Notably, ∼70% of the MAP and HR predictions fell within two SEM of the experimental data, indicating that the model predictions were generally indistinguishable from the experimentally measured mean values. In addition, the model yielded a relatively low, non-systematic bias for MAP, with MBE ranging from −5.58 to 2.68 mmHg across the three studies. In contrast, the model underpredicted HR, with MBE ranging from −0.47 to −7.24 beats/min. Figure 3 (shaded areas) also shows the ranges of the model-predicted MAP and HR based on 1,000 simulations, using parameter values of the PD model from within the 95% confidence interval of their estimated mean values. In these simulations, the peak MAP values fell between 90 and 143 mmHg (nominal model: ∼120 mmHg) and the peak HR values fell between 68 and 92 beats/min (nominal model: ∼80 beats/min). At the end of the 60-min ketamine administration in Studies *1* and *3*, MAP fell between 84 and 136 mmHg (nominal model: ∼97 mmHg) and HR fell between 65 and 85 beats/min (nominal model: ∼70 beats/min). Overall, the model reasonably captured the effect of ketamine on cardiovascular dynamics in healthy individuals.

### 3.3 Simulation results

**FIGURE 4:**
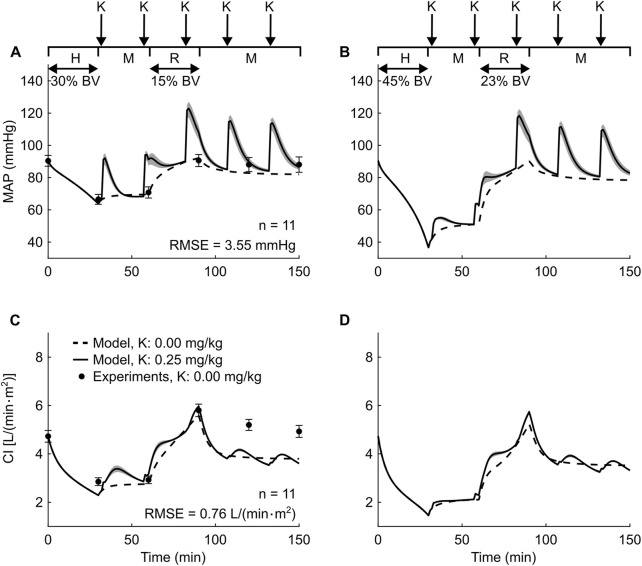
Results of simulations performed to investigate the effect of ketamine on mean arterial pressure (MAP) **(A,B)** and cardiac index (CI) **(C,D)** following hemorrhage. The left panel **(A,C)** shows the results for a 30% blood volume (BV) loss and the right panel **(B,D)** shows the results for a 45% BV loss. The timeline at the top of each panel illustrates the experimental protocol, wherein the arrows represent bolus ketamine (K), H represents the hemorrhage period, M denotes the monitoring period, and R represents the resuscitation period. The solid line denotes the model predictions for a dose of 0.25 mg/kg, while the shaded area represents the range of the predicted vital signs, with the lower bound representing a ketamine dose of 0.20 mg/kg and the upper bound a dose of 0.30 mg/kg, for a typical 70-kg human. Error bars denote two standard errors of the mean. RMSE: root mean square error.

We investigated the effect of ketamine on MAP and CI during hemorrhage and resuscitation. Specifically, we simulated two scenarios, each with and without ketamine: a moderate hemorrhage (30% of total blood volume; Figures 4A,C) based on Urbano et al. (2012) and a severe hemorrhage (45% of total blood volume; Figures 4B,D), followed by fluid resuscitation of 15% and 23% of total blood volume, respectively. Starting at 2 min after the end of hemorrhage and four more times at 25-min intervals, we simulated ketamine administration in doses ranging from 0.20 to 0.30 mg/kg. Without ketamine administration, as in the experimental study involving moderate hemorrhage (Figures 4A,C, solid circles) (Urbano et al., 2012), our model predictions showed that both MAP and CI initially decreased by ∼30% and ∼52% of their baseline values, i.e., the pre-hemorrhage, steady-state values, respectively, and then returned back to their baseline values after resuscitation (Figures 4A,C, dashed lines). We obtained reasonable RMSEs of 3.55 mmHg for MAP and of 0.76 L/(min·m^2^) for CI between the model predictions and the corresponding experimental data. For the second scenario involving severe hemorrhage for which we did not have experimental data, we observed similar trends for MAP and CI (Figures 4B,D, dashed lines).

With ketamine administration, during both moderate and severe hemorrhage, we observed that the predicted values for MAP and CI increased rapidly (in 1–5 min) with each ketamine dose (Figure 4, solid lines) and then returned to their corresponding predicted values without ketamine administration (Figure 4, dashed lines) within ∼25 min, before a subsequent ketamine dose. Interestingly, the magnitude of the ketamine-induced increases in MAP and CI decreased with hemorrhage severity. For instance, compared to no ketamine administration (Figure 4A, dashed line), when the same ketamine dose of 0.25 mg/kg for a 70-kg human was first provided at 32 min, MAP increased by a maximum of ∼30% of its baseline value for the moderate hemorrhage case (Figure 4A, solid line) but only by ∼11% for the severe hemorrhage case (Figure 4B, solid line). Similarly, CI increased by ∼15% of its baseline value for moderate hemorrhage (Figure 4C, solid line) and by only ∼4% for severe hemorrhage (Figure 4D, solid line). This is consistent with the predictions of MAP in Figure 3B, where after ketamine administration of the same dose provided at 0 min, we observed an ever larger maximum increase of 40% of its baseline value in the absence of hemorrhage. However, for CI, we observed similar increases with moderate or no hemorrhage after ketamine administration. Finally, we observed that variations in ketamine dose between 0.2 and 0.3 mg/kg resulted in minor changes in the values of the vital signs (Figure 4, shaded areas).

## 4 Discussion

In future large-scale conflicts with a near-peer adversary, combat medics will face numerous challenges, including high casualty rates, limited resources for treating causalities, and delayed evacuation. In such a mass-casualty scenario, AI algorithms that allow for rapid decision-making and increased capacity will be essential in assisting combat medics to care for the large number of casualties in a resource-constrained environment (Jin et al., 2024). To develop this capability, we will need mathematical models that can accurately predict the human physiological response to major battlefield injuries and associated treatments in order to generate large amounts of synthetic data to train the AI algorithms and evaluate their performance. To this end, we previously developed and validated the CR model, which can simulate a wide range of hemorrhagic injuries and the associated fluid resuscitation treatments using six different fluid types (Jin et al., 2023; Kurian et al., 2025). Because most trauma casualties are treated with analgesics, such as fentanyl or ketamine, which affect the cardio-respiratory system, to develop a capability that represents the actual operational environment we must incorporate the effects of these drugs into the CR model (Shackelford et al., 2015; Schauer et al., 2017; Vankawala et al., 2021; Abuhelwa et al., 2022). Here, we extended the CR model to incorporate the effect of ketamine, the recommended analgesic for hemorrhagic shock, on the vital signs (MAP, HR, and CO) of trauma casualties. By adding this capability to the model, we can now simulate a wider range of treatment scenarios after hemorrhagic injury.

We assessed the performance of the extended CR model by comparing its predictions following racemic ketamine administration at analgesic doses with the time course of vital signs from three experimental studies in healthy individuals (*Studies 1–3*). On average, the model captured the changes in the vital signs reasonably well, with RMSEs for MAP (6.17 mmHg) and HR (7.51 beats/min) within 7%–10% of their baseline values (Hall and Hall, 2020). These RMSEs were slightly larger than the accuracy of representative Food and Drug Administration-cleared vital-sign monitors. For example, the standard deviation of HR and SBP for the ZOLL Propaq M monitor is 3 beats/min and 3 mmHg, respectively (ZOLL Medical Corporation, 2016). However, such RMSEs are acceptable because the primary goal of synthetic data sets is to capture physiological trends to develop AI algorithms rather than to replicate the precise responses of any one specific individual. We could not find experimental studies [apart from the one used to develop the PD model of CO (Kamp et al., 2021)] that reported the effect of racemic ketamine administration on CO in healthy individuals or those suffering from hemorrhagic injuries. However, in our simulations, CO demonstrated the same trends as MAP and HR, i.e., in healthy individuals it increased ∼10% above its baseline value upon ketamine administration and returned to its baseline value within 25 min.

Pain management is a crucial aspect of combat casualty treatment, where over 70% receive either one or multiple analgesic drugs (Shackelford et al., 2015; Schauer et al., 2017). While highly effective in relieving pain, both opioid and non-opioid analgesics have detrimental side effects on the cardio-respiratory system and their vital-sign response. For example, administration of synthetic opioids, such as alfentanil, fentanyl, and sufentanil, has been shown to reduce the ventilatory rates of healthy individuals and of those recovering from surgery by at least 25% of their baseline values (Van der Auwera et al., 1987; Mildh et al., 2001; Dahan et al., 2005). Similarly, morphine, a natural opioid with lower potency, has also been shown to reduce the CO of critically ill patients by about 20% of their baseline values (Rouby et al., 1981; Dahan et al., 2005). In addition, the well-established addictive properties of opioids make them less suitable for long-term use (Buckenmaier and Griffith, 2010). Similarly, administration of non-opioid analgesics in the form of imidazolines, such as clonidine, has been shown to reduce MAP by ∼15% of its baseline value in healthy individuals (Hall et al., 2001). In contrast, administration of ketamine, when provided in doses of less than 0.5 mg/kg, has been known to induce vital-sign changes that could be potentially favorable in a hemorrhagic-injury scenario (Xiang et al., 2020). For example, as corroborated by previous experimental studies of healthy individuals (Kochs et al., 1991; Långsjö et al., 2003), our predictions showed that the administration of analgesic doses of ketamine elevated MAP by ∼40% and HR by ∼15% of their baseline values in less than 2 min, while preserving respiratory function (e.g., minute ventilation changed by <3%). In terms of MAP and low oxygen perfusion, such cardio-respiratory responses are beneficial for hemorrhagic injuries, which is likely why ketamine is recommended over other drugs in its class after a traumatic event (Committee on Tactical Combat Casualty Care, 2024).

Because we could not identify clinical data from humans or studies in large animals that reported the effect of analgesic ketamine on vital signs after hemorrhagic injury, we investigated this effect through simulations using the CR model. Interestingly, we observed that ketamine-induced increases in MAP and CO decreased as hemorrhage severity increased (Figure 4). Such a diminishing effect of ketamine on MAP has been previously reported in humans suffering from trauma (not necessarily involving hemorrhage). For example, Miller et al. (2016) reported that in patients intubated in a pre-hospital environment due to trauma or illness, including head injury, burns, and respiratory failure, ketamine administration increased their systolic blood pressure by an average of 17 mmHg when patients had a minor injury or illness (shock index <0.9) but by only 3 mmHg when they had a major illness or injury (shock index ≥0.9). While its exact neurological mechanisms are complex (Mion and Villevieille, 2013), we do know that ketamine activates the sympathetic nervous system (Peltoniemi et al., 2016; Goddard et al., 2021; Keith et al., 2022), which in turn plays a key role in maintaining homeostasis during hemorrhagic injuries (Chien, 1967; Carrara et al., 2018; Ranjan and Gulati, 2023). However, our model predictions showed that during severe hemorrhage (i.e., loss of over 45% of total blood volume), the sympathetic activity reached saturation (Batchinsky et al., 2007; Carrara et al., 2018), and further ketamine administration did not lead to any noticeable increase in the vital signs. Thus, based on our simulations, we hypothesize that ketamine administration could potentially be beneficial when treating mild to moderate, but not severe hemorrhagic injuries.

In recent years, PK-PD models have become an essential component of the drug development process (Vinks and Barrett, 2021; Hong-can, 2025). These models can provide valuable information regarding the distribution and accumulation of different drugs based on their regimen and how these drugs affect physiological responses, such as blood pressure and HR, even when their mechanisms are not fully known. As such, these models have been instrumental in identifying safe dose regimens and establishing guidelines for the proper use of many drugs. For example, while the exact mechanisms of mood-stabilizing drugs, such as lithium, are not well understood, PK-PD models of lithium have served as tools to optimize lithium dosing for individual patients by considering factors like renal function and weight (Landersdorfer et al., 2017). However, at the same time, developing PK-PD models requires a large amount of clinical or experimental data, and the models are specific to the particular pathology or disease data on which they were trained, limiting their generalizability (Steele et al., 2015). Conversely, mechanistic models, such as the CR model, incorporate known biological mechanisms and interactions and can represent various injuries and illnesses, but cannot represent the effect of drugs if their mechanisms are not known (Kulesza et al., 2024; Kurian et al., 2025). By integrating PK-PD models of ketamine into our CR model, we combined the capabilities of these two model types, which allowed us to specifically investigate the role of ketamine during a hemorrhagic injury. In this manner, we could potentially combine existing PK-PD models of other drugs commonly used to treat battlefield injuries, such as tranexamic acid and midazolam (Haschke et al., 2010; Li et al., 2021), within the CR model to determine how they affect vital signs. We could also potentially test the effects of administering different drug combinations and dosing regimens.

We are aware of only one other model, the Pulse Physiology Engine, that has the capability to model the effects of both hemorrhage and ketamine administration on vital signs (Clipp et al., 2016; Bray et al., 2019). While the Pulse model predictions of vital-sign changes resulting from hemorrhage have been quantitatively validated, their predictions of the effect of ketamine in healthy individuals have only been qualitatively assessed. In contrast, we have previously quantitatively validated the CR model for a variety of hemorrhagic injury scenarios (Jin et al., 2023) and here we present quantitative validation for the effect of ketamine on healthy individuals for a range of ketamine doses (0.25–0.91 mg/kg), administration rates (0.01–0.25 mg/min), and regimens (slow and bolus infusion). In addition, we also generated a hypothesis regarding the diminishing effects of ketamine on MAP and CO with increasing hemorrhage severity, which needs to be experimentally tested in large-animal studies under hemorrhagic conditions to substantiate the model’s potential benefit in predicting the physiological responses to ketamine in hemorrhagic shock states.

Although the extended CR model generally captured the variations in vital signs during ketamine administration, it does have a few limitations, primarily due to the simplifying assumptions we made during model development. First, our assumption that ketamine PK/PD in healthy individuals would be the same for those with hemorrhagic injury may be inaccurate. However, it was necessary due to the lack of clinical or experimental data involving both hemorrhage and ketamine administration. In fact, hemorrhagic shock has been shown to alter the PK/PD of other analgesics, such as fentanyl (Bienert et al., 2020; Egan and Johnson, 2020), and therefore, it is reasonable to expect similar effects in ketamine PK/PD, as well. Hence, our assumption could lead to over- or under-estimation of changes in vital signs induced by ketamine administration following a hemorrhagic event. Second, we did not model the effect of ketamine on ventilatory responses, such as minute ventilation and respiratory rate. While a few studies investigated the effect of ketamine on respiratory rate, they report contradictory results in the magnitude and directionality of the effect (Domino et al., 1965; Morel et al., 1986; Långsjö et al., 2003), although the prevailing consensus is that administration of ketamine preserves ventilatory rates (Haas and Harper, 1992; Peltoniemi et al., 2016; Butterworth et al., 2022). Third, the CR model does not account for the mechanisms by which pain and distress affect the vital signs and how ketamine might alter these effects, because such mechanisms are currently unknown (Tousignant-Laflamme et al., 2005; Jin et al., 2023). Finally, while there are multiple administration routes for ketamine, including intravenous, intranasal, intramuscular, and subcutaneous, our PK model only considered ketamine kinetics via intravenous administration because it is the recommended route in battlefield trauma (Committee on Tactical Combat Casualty Care, 2024). If necessary, in the future, we can further extend the CR model by incorporating other ketamine administration routes using the corresponding ketamine PK models.

In summary, we extended and validated our previously developed CR model to predict the temporal change in vital signs caused by administration of ketamine. This new capability will enhance the range of treatment scenarios of the CR model and allow us to generate synthetic trauma casualty data that include hemorrhagic injury, placement of tourniquet, fluid resuscitation with saline, blood, and blood products, and administration of pain medication. Such a capability will also allow us to generate more diverse trauma data sets to develop AI algorithms.

## Data Availability

The original contributions presented in the study are included in the article/supplementary material, further inquiries can be directed to the corresponding author.
